# Barriers and facilitators to the uptake of Test and Treat in Mozambique: A qualitative study on patient and provider perceptions

**DOI:** 10.1371/journal.pone.0205919

**Published:** 2018-12-26

**Authors:** Pedroso Nhassengo, Fabian Cataldo, Amilcar Magaço, Risa M. Hoffman, Lucas Nerua, Mohomede Saide, Rosa Cuco, Roxanne Hoek, Francisco Mbofana, Aleny Couto, Eduardo Gudo, Sergio Chicumbe, Kathryn Dovel

**Affiliations:** 1 Health System Research Sector, National Institute of Health, Maputo, Mozambique; 2 Institute for Global Health and Development, Queen Margaret University, Edinburgh, United Kingdom; 3 David Geffen School of Medicine, University of California Los Angeles, Los Angeles, California, United States of America; 4 National STI-HIV/AIDS Program, Ministry of Health, Maputo, Mozambique; 5 National Council against HIV/AIDS, Maputo, Mozambique; 6 Research Department, Partners in Hope, Lilongwe, Malawi; Oregon State University, UNITED STATES

## Abstract

**Background:**

In mid-2016, Mozambique began phased implementation of the ‘Test-and-Treat’ policy, which enrolls HIV positive clients into antiretroviral treatment (ART) immediately regardless of CD4 cell count or disease stage. Novel insights into barriers and facilitators to ART initiation among healthy clients are needed to improve implementation of Test and Treat.

**Methods and findings:**

A cross-sectional qualitative study was conducted across 10 health facilities in Mozambique. In-depth interviews (IDIs) were conducted with HIV-positive clients (60 who initiated/20 who did not initiate ART within Test and Treat) and 9 focus group discussion (FGDs) were conducted with health care workers (HCWs; n = 53). Data were analyzed using deductive and inductive analysis strategies. Barriers to ART initiation included: (1) feeling ‘healthy’; (2) not prepared to start ART for life; (3) concerns about ART side effects; (4) fear of HIV disclosure and discrimination; (5) poor interactions with HCWs; (6) limited privacy at health facilities; and (7) perceptions of long wait times. Facilitators included the motivation to stay healthy and to take care of dependents, as well as new models of ART services such as adaptation of counseling to clients’ specific needs, efficient patient flow, and integrated HIV/primary care services.

**Conclusions:**

ART initiation may be difficult for healthy clients in the context of Test-and-Treat. Specific strategies to engage this population are needed. Strategies could include targeted support for clients, community sensitization on the benefits of early ART initiation, client-centered approaches to patient care, and improved efficiency through multi-month scripting and increased workforce.

## Introduction

In 2015, the World Health Organization (WHO) announced new universal treatment guidelines for antiretroviral treatment (ART), which support initiation of ART for all individuals living with HIV, independent of their immunologic or clinical status.[1] Since then, countries throughout sub-Saharan Africa have adopted the “Universal Test-and-Treat” (Test and Treat) strategy.[2] The strategy is expected to contribute to improved client outcomes and attaining UNAIDS 90-90-90 treatment targets, specifically the ART coverage target.[3] Despite rapid advances in the implementation of Test and Treat, to date, there are limited data on barriers and facilitators to ART initiation under the new policy, particularly among non-pregnant and non-breastfeeding populations who are, for the first time, eligible for ART initiation when asymptomatic at the time of HIV diagnosis (i.e., healthy clients). Limited research has been conducted in Mozambique to understand barriers and facilitators to ART initiation.[4–11]

Previous research in sub-Saharan Africa identified multiple barriers to ART initiation for adults eligible for treatment based on clinical criteria, such as CD4 count or WHO stage.[12–15] Dominant barriers include: concerns related to stigma, confidentiality, and privacy whilst accessing HIV services; negative treatment by clinical staff; conflicting demands on clients’ time; distance to the health facility; poor knowledge of ART regimens; and fear of side effects.[15–20] Another body of literature on Option B+, whereby universal treatment is available for all pregnant and breastfeeding women regardless of CD4 count, may provide insight on the experiences of healthy populations. Pregnant and/or breastfeeding women eligible for treatment under Option B+ report the following barriers to ART initiation: not prepared to start lifelong treatment; fearing partner response to a positive status; fear of disclosure; and fear of side effects.[21–23] While these findings provide insight to healthy populations, they may not apply to healthy, non-pregnant clients. Non-pregnant/breastfeeding HIV positive clients who feel healthy may not perceive or understand the need to start ART immediately; they also may not have the additional motivation of preventing mother-to-child-transmission experienced by pregnant/breastfeeding women.

We performed a qualitative study to assess barriers and facilitators to ART initiation among healthy clients eligible for ART under the new Test and Treat policy in Mozambique. The study includes perspectives from both providers and clients. This is one of the first studies in sub-Saharan Africa to explore barriers to ART uptake among asymptomatic non-pregnant/breastfeeding populations in the context of Test and Treat.

## Methods

### Setting

In Mozambique, a country of 27 million people, an estimated 13.2% of adults (aged 15 to 49 years) are living with HIV.[24] To date, approximately 56% of clients eligible for ART have initiated treatment.[25] In August 2016 Mozambique began a phased implementation of Test and Treat, known locally as *Testar e Iniciar* (“Test and Start”), which intends to enroll all HIV positive clients into care and start ART within fifteen days of a positive HIV diagnosis. Mozambique has taken a human rights approach to linkage to care. While linkage is actively facilitated, clients who test HIV positive and are not ready to initiate ART can remain anonymous, allowing positive clients to ‘opt-in’ by presenting themselves for ART rather than being actively traced. Clients may enroll into ART program to receive support for their HIV positive status, such as counseling and support groups, and decline treatment initiation.

### Study sites

**Fig 1:**
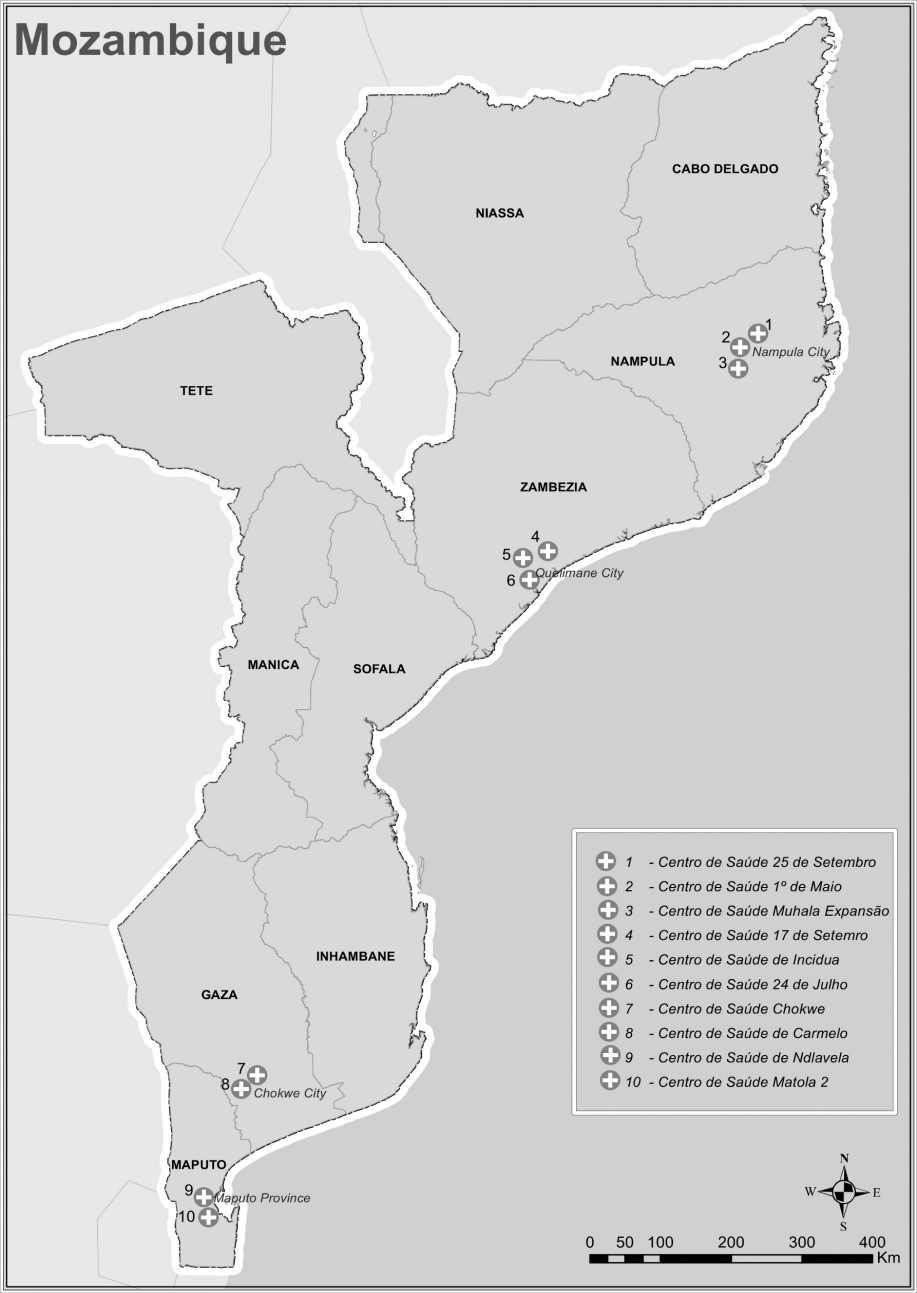
Map of study sites.

Study participants were recruited from 10 health facilities located in urban and peri-urban areas across 4 provinces in the Northern, Central, and Southern regions of the country. Sites were eligible if they had a medium to large ART cohort size (defined as > 2000 patients currently on ART) and an active electronic patients tracking system (EPTS) in place 12 months before the Test and Treat policy. All included sites began the Test and Treat rollout in August 2016. Provincial, central and private hospitals were excluded because of their non-representative characteristics of the primary level of care and HIV services (in relation to staffing, service provision and levels of technical expertise). Since multiple sites from the same province met inclusion criteria, we randomly selected a geographical region within each province (see Fig 1). Eligible sites from the same geographical region were grouped together and numbered. Through consecutive randomization processes, 12 potential sites were identified. Two sites were excluded due to concerns around accessibility and low number of new enrollees during the study period.

### Study participants and data collection

Focus group discussions (FGDs) (n = 9) were conducted with health care workers (HCWs) at study sites. A total of 53 HCWs participated. Four of the 9 FGDs were conducted with health care providers who directly provided ART care (doctors, clinical officers, nurses, maternal and child health nurses, laboratory, and pharmacy staff). The other 5 FGDs were conducted with supporting staff who were engaged in HIV care services (HIV counselors, community health workers called “*Agente Polivalente Elementary”*, and trained peer educators). HCWs were eligible for the study if they had been actively engaged in HIV/ART services for at least one year. Discussions focused on HCW observations and perceptions of barriers and facilitators to ART initiation under Test and Treat. We specifically asked if HCWs believed there barriers and facilitators differed for healthy clients under the new policy as compared to clients eligible for ART under older eligibility criteria that relied on CD4 count and/or clinical stage.

**Fig 2:**
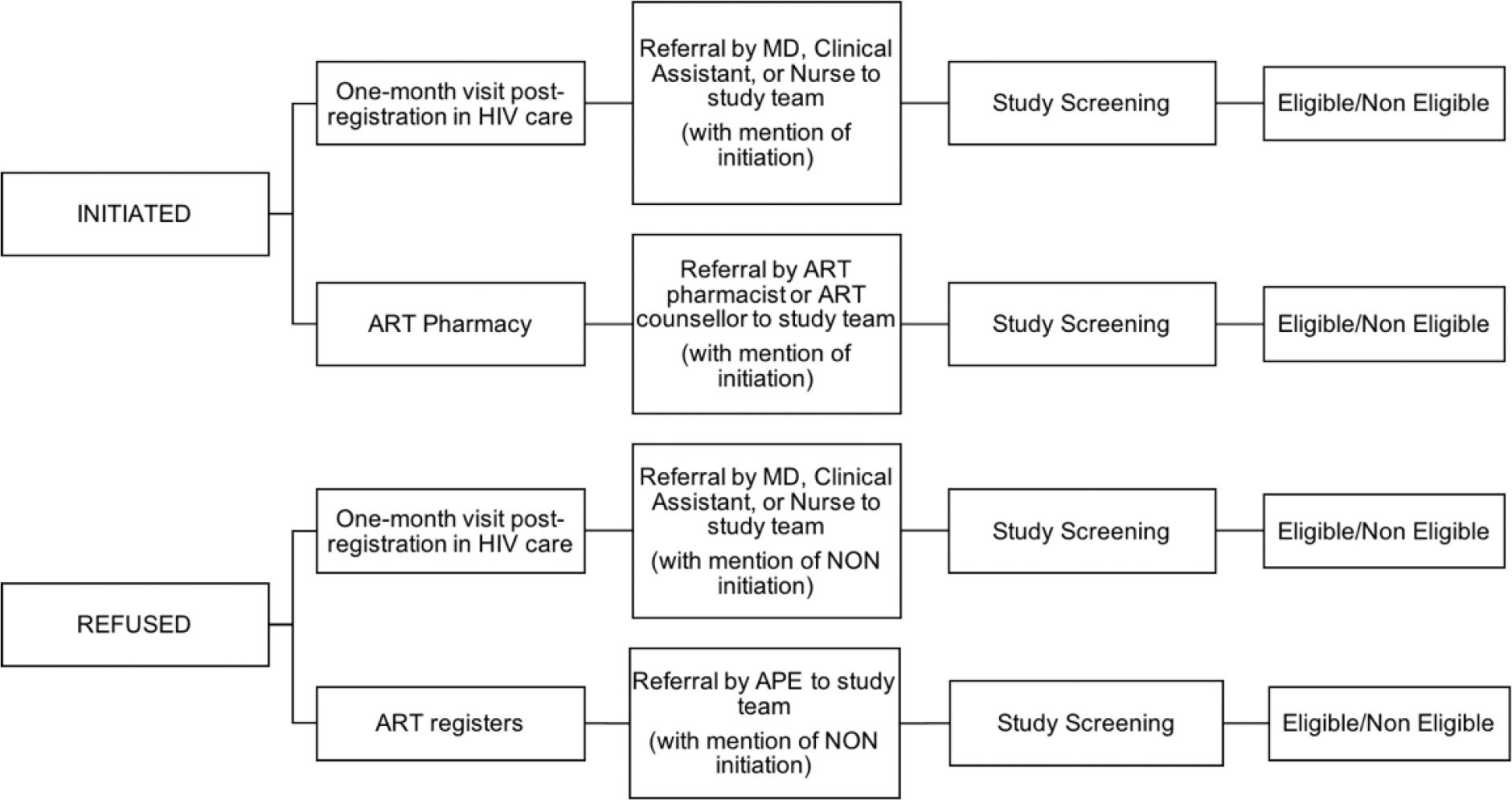
Study recruitment process and screening.

In-depth interviews (IDIs) were conducted with 80 people (43 females and 37 males). Eligibility criteria included: (1) non-pregnant/breastfeeding; (2) adult (15 years of age and older); (3) newly diagnosed HIV-positive between August 2016 –February 2017, after the implementation of Test and Treat; and (4) asymptomatic at the time of diagnosis (i.e., not requiring ART initiation due to acute illness or opportunistic infection. We enrolled two groups of individuals who received an HIV positive test under Test and Treat and categorized them based on whether they initiated ART within 14 days of diagnosis (based on government guidelines recommending initiation within a 14 day window): (1) those who initiated ART within 14 days after receiving an HIV positive diagnosis (n = 60) (‘initiates’) and (2) those who were enrolled into care but did not initiate ART within 15 days (n = 20) (‘non-initiates’). New ART initiates were recruited after ART uptake and counseling sessions. Non-initiates were recruited from HIV service clinics that provide support for people living with HIV or from referrals through community health workers. All clients were screened through the standard study screening procedure (see Fig 2). The interview guide was informed by existing literature [10, 11, 15, 18, 19] and the socioecological model that provides a platform to examine the multiple and dynamic levels within individuals, communities, and society that influence ART initiation [26]. The interview guide included four domains: sociodemographic data; experience with HIV diagnosis and HIV services at their local health facility; perceptions of ART; and perceived barriers and facilitators to their initiation of ART, drawing specifically on barriers and facilitators at multiple levels of the socioecological model.

All data were collected by trained, local research assistants who were experienced in qualitative research in the field of HIV and health services. Data collection tools were developed based on the outcomes of similar studies carried out in the region and open-ended questions were predominant. Probing technique was applied for following up and clarification as needed. In-depth interviews lasted approximately 40 minutes while focus group discussions lasted approximately 80 minutes.

### Analysis

All FGDs and IDIs were recorded and transcribed verbatim in Portuguese and translated into English. Analytical themes and codes were derived from the study objectives around facilitators and barriers of ART uptake and retention in care.[27] Transcripts were analyzed using *NVivo11* software to extract and sort interview text into single-file statements with the same code from all interviews.[28] Deductive and inductive coding was conducted independently by PN, LN, MS, FC, and AM.[29] Codes were compared for consistency and all discrepancies resolved across the five investigators.

### Ethical considerations

This study was approved by the Institutional Ethics Committee of the Instituto Nacional de Saúde (National Institute of Health) in Maputo, Mozambique (ref. no. 025/CIBS-INS/2017) and the Institutional Review Board at the University of California, Los Angeles, USA (approval no. 17–001182). Based on Mozambique law, no parent/guardian consent was needed for participants. This was approved by local ethics review committees. Further, while our eligibility criteria was 15 years of age or older, we did not enroll anyone below 18 years (based on who presented as a new HIV positive client during the study period). The study was performed in accordance with the study protocol, the Declaration of Helsinki (October 2013) and the WHO Handbook for Good Clinical Research Practice (July 2002). Participants were not paid for their participation in the study. All study participants completed a written informed consent prior to enrollment in the study. Thumb prints were taken for respondents who were unable to write.

## Results

### Socio demographic data

Sociodemographic data of HIV-infected participants in IDIs are described in Table 1. Respondents included 43 females and 37 males. Median age was 37 (IQR, 28–43). Thirty-seven respondents had known their HIV status for 1–3 months, and 43 for more than 3 months. Education levels varied, with most completing at least primary school. Most interviewees received some income from formal or informal work including subsistence agriculture (83%). Average travel time to health facilities ranged from 30 minutes to 2 hours, with about half of the interviewees (51%) requiring less than 30 minutes to reach a health facility. More than half of the respondents (57%) had disclosed their HIV status to their spouse or partner, and more than half stated that they knew their partner’s serostatus (55%).

**Table 1:** Socio-demographic data–individuals living with HIV.

	Initiates (*n* = 60)	Non-initiates (*n* = 20)	Total (*N* = 80)
**Median age (IQR)**	37	38	**37 (28–43)**
**Male** **Female**	24 (40%) 36 (60%)	13 (65%) 7 (35%)	**37 (46%)** **43 (54%)**
**Mean n. of children** (Range)	2 (0–2)	1 (0–1)	**1 (0–2)**
**Education level completed**			
*Primary*	27 (45%)	7 (35%)	**34 (42.5%)**
*Secondary/Higher*	22 (37%)	11 (55%)	**33 (41%)**
*No education*	11 (13%)	2 (10%)	**13 (16%)**
**Occupation**			
*Formal/Informal work*	52 (87%)	15 (75%)	**67 (84%)**
*No work/Student*	8 (13%)	5 (25%)	**13 (16%)**
**Time knowing HIV+ serostatus**			
*1–3 Months*	32	9	**41**
*3–6 Months*	28	11	**39**
**Average travel time to clinic**			
*< 30 Min*	30	11	**41**
*30 Min-2h*	25	9	**34**
*> 2h*	5	0	**5**
**Self-assessment of health status**			
*Very good/Excellent*	33 (55%)	8 (40%)	**41 (51%)**
*Good*	26 (43%)	10 (50%)	**36 (45%)**
*Poor*	1 (1%)	2 (10%)	**3 (3%)**
**Disclosed to partner**	36 (60%)	10 (50%)	**46 (57%)**
**Know partner status**	34 (56%)	10 (50%)	**44 (55%)**
**Partner status**			
*HIV +*	25 (42%)	9 (45%)	**34 (42.5%)**
*HIV -*	9 (15%)	1 (5%)	**10 (12.5%)**
*Refuse to disclose*	4 (7%)	1 (5%)	**5 (6%)**
*Do not know*	22 (36%)	9 (45%)	**31 (39%)**
**Total informants by Province**			
*Maputo*	11	5	**16**
*Gaza*	20	3	**23**
*Zambézia*	13	3	**16**
*Nampula*	16	9	**25**

Sociodemographic data of providers participating in FGDs are described in Table 2. Respondents included 20 females and 33 males. Median age was 36 years (IQR 28–40) and median years working in HIV services was 4 years (IQR 2–7).

**Table 2:** Socio-demographic data–Health care providers participating in FGDs.

	Maputo (n = 12)	Gaza (n = 14)	Zambézia (n = 8)	Nampula (n = 19)	Total (N = 53)
**Median age** (IQR)	**29** (28–39)	**36** (29–39)	**36** (26–40)	**36** (30–44)	**36** (28–40)
**Male** **Female**	3 9	8 6	8 0	14 5	**33 (62%)** **20 (38%)**
**Occupation**					** **
Health facility clinical staff (doctor, clinical officer, nurse, pharmacist, lab technician, etc.)	5	8	4	9	**26**
Supporting staff (counselors, community health workers)	7	6	4	10	**27**
**Median years working in HIV services** (IQR)	**2** (1–5)	**4.5** (4–7)	**3** (3–9)	3 (1–6)	**4 (2–7)**

### Barriers to initiating ART

Both HCWs and clients identified a range of barriers to ART initiation.

#### Client acceptance of HIV status and ART

Feeling ‘healthy’ was described by health providers (8/9 FGDs) and some of the clients (7/80) as one of the main reasons for not believing a positive test result. One female client who refused to start ART described her “disbelief” at receiving a positive HIV diagnosis despite feeling well: *“I didn't believe it* [HIV test result], *I’m fine*, *I don’t feel anything”* (Female client, Non-initiate, Maputo). Another client stated: *“I thought they* [HCWs] *were playing with me”* (Male client, Non-initiate, Gaza).

Feeling ‘healthy’ and being asymptomatic was also cited by most providers (9/9 FGDs), and some clients (7/80), as an important barrier to starting ART. One of the HCWs in Nampula explained that *“patients say*: *I am not yet prepared because I am not yet sick*, *I am not yet sick*, *I cannot accept to start with the treatment today”* (female HCW, support staff, Nampula). Another HCW described:

*“A person*, *as long as he has no symptoms*, *having been tested and diagnosed*, *to say he has to start ART does not seem to be a reality; the person wants to believe that he is ill when he is in bed*, *while he is still healthy does not feel like he is really sick”* (male HCW, support staff, Gaza).

Lack of information about ART was cited by both health providers and clients as an important barrier to starting ART. Some clients complained about having to start medication without fully understanding adherence requirements and how long they needed to take treatment. Others explained they were not aware of the new Test and Treat policy and felt confused and unprepared to start treatment immediately after a positive test.

*“They* [HCWs] *were not very explicit*, *they didn’t explain much*. *They just started the process and said tomorrow you should come back to pick up these medicines*, *I didn’t know what medicines they were talking about*.*”* (Male client, Non-initiate, Nampula)

*“Every patient resists”* added one of the HCWs in Zambézia, who explained that clients may not understand the reason for starting ART and the necessity to be on any treatment. Another HCW described the difficulties in starting ART immediately after the HIV test:

*“It is hard for them* [clients] *to suddenly start the treatment immediately after the results*, *some who are supposed to benefit from this treatment may not understand* […] *for some people it is so sudden and immediate” “Some people when they are tested are very skeptical to start on the very same day”* (male HCW, support staff, Gaza).

Several clients (26/80) expressed being reluctant to start ART ‘for life’. One of the women in Gaza said: *“pills for the rest of life is worrying”* (Female client, Initiate, Gaza). One woman in Maputo who refused to start ART described the challenge of embarking on lifelong therapy:

*“I will have to take pills forever… Tablets forever*, *my God I never thought that would happen to me*, *it’s unbearable to take pills the whole life*. *[…] taking pills for life every day is complicated*, *very complicated”* (Female client, Non-initiate, Maputo).

#### Perceived side effects

Most clients (59/80) reported that feeling dizzy and unwell could lead them to stop taking ART: “*They* [ARVs] *torture me*, *I feel bad when I take more”* said a woman who had recently initiated ART in Gaza (Female client, Initiate, Gaza). Another woman attests to the personal challenges of starting ART: *“on the first day*, *the reaction is very strong and you can even quit if you are not strong and determined”* (Female client, Initiate, Nampula).

Concern about side effects was also cited as a key barrier during all discussions with HCWs, who described their clients’ fear of possible harmful reactions:

*“She* [patient] *was doing the treatment here but had severe reactions to the drugs and her family didn’t understand what was going on and they thought that it was witchcraft”* (male HCW, support staff, Zambézia).

Some health providers also pointed at widespread negative beliefs around HIV and ART, whereas clients described ART as a poison purposefully dispensed by HCWs. One provider described an interaction with a client who was trying to decide to whether to take treatment or not:

*“[the patient said]*: *‘My mother said don’t take it* [ART]. *Let’s do the family treatment* [homemade herbal medication] *because it* [ART] *will kill you*, *those people* [HCWs] *want to kill you’”* (female HCW, clinical staff, Nampula).

#### Disclosure and partner involvement

Health providers and clients described the challenge of disclosure for individuals who recently tested HIV positive: *“I do not trust other people”*, said one female client who initiated treatment in Gaza. Fear of discrimination, being stigmatized, and lack of trust towards sexual partners and health providers were cited by the majority of clients (59/80) as the main reason for not disclosing their serostatus to other individuals, including family members, friends, neighbors and the larger community; *“When you tell them* [other individuals], *they start to discriminate and stigmatize you*, *saying you are taking antiretrovirals”* (Female client, Initiate, Gaza).

One of the HCWs described their clients’ fear of disclosing in relation to the pervasive stigma around HIV transmission:

*“*[…] *what comes to their* [clients’] *mind is that being HIV positive is equal to having had promiscuous conduct*, *bad conduct that the society doesn’t approve of”* (male HCW, support staff, Gaza).

HCWs (7/9 FGDs) indicated that some of the women who refused to start ART needed to seek consent from their partner in order to start treatment. The low level of disclosure within couples limited partner involvement. Several women feared that disclosing their serostatus or openly discussing ART initiation with their partner would negatively impact their relationship and result in rejection from their partner.

*“Wives do not reveal [their HIV status] because they fear that the partner can beat them or even get divorced because nowadays when the men know about it*, *they abandon the family”* (female, support staff, Zambézia).

One of the women who initiated ART in Gaza described the changes in her relationship after sharing that she is living with HIV: “*I realized that nothing was worthwhile looking at the way he* [partner] *started talking to me*. […] *He stopped treating me like a lady and things got worse day in and day out”* (Female client, Initiate, Gaza).

In Maputo, one of the women interviewed who refused to start ART narrated some of her challenges in sharing her serostatus with her partner:

*“I left [the facility] with the determination that I will tell him* [partner] *that* [I am HIV positive] *but when I arrived I didn’t have the courage* […] *He will put the blame on me*. […] *I really want to* [start ART] *but I cannot start the treatment without my partner I live with*. *Imagine if I start the treatment I have to take pills where am I going to hide the pills*? *In our house*, *there is no place to hide*. […] *I don’t want to start treatment alone* […] *it’s as if I were betraying him*. […] *The biggest cause for me not to start treatment is my partner*, *he doesn’t know* [that I am living with HIV]*; I don’t know how to tell him*. (Female client, non-initiate, Maputo)

#### Interactions with providers and privacy at health facilities

Some HIV-infected clients (30/80) described negative interactions with HCWs. Clients attributed self-transfers to other health facilities or the desire to stop ART to the perceived negative attitudes of HCWs. Some of the clients (15/60) who had recently initiated ART perceived mistreatment by HCWs as having a negative impact on every aspect of their clinical visits:

*“They* [doctors and nurses] *treat us as if we are mad people*. *And most of the time people who are infected tend to lose their hope in taking their life forward*” (Female client, Initiate, Maputo).

Due to limited privacy at health facilities, clients who felt healthy tended to avoid ART clinics because they were concerned about being seen by relatives, friends, or neighbors. HCWs (9/9 FGDs) reported that lack of privacy at health facilities hinders ART uptake and retention in care. Some clients (27/80) also raised privacy as a barrier, some traveling long distances to seek HIV care where minimum privacy could be ensured; HCWs indicated that clients who travel long distances for HIV care are more likely to default due to limited transport money and other expenses related to accessing care. One of the women in Gaza mentioned that *“there is no privacy*, *even when they* [HCWs] *give a prescription*, *because of the call* [in public], *people know that so-and-so comes to get pills* [ART]” (Female client, Initiate, Gaza). Some initial HIV counseling sessions for newly diagnosed individuals are set up as ‘group counseling’, and clients reported this as an additional deterrent due to lack of privacy and confidentiality.

#### Long wait times

The perception of the time required for HIV services negatively impacted clients’ ability to initiate and continue ART, particularly for clients who fear disclosure or fear to lose their job for absenteeism. One male client who refused to initiate said: *“This* [wait time] *makes it a bit difficult because I live based on small business*, *if I’m here for 30 minutes*, *this is wasting my time”* (Male client, Non-initiate, Gaza).

One of the HCWs raised that it may seem impossible for some clients to take time off to attend monthly clinic visits:

*“The person* [client] *says […] I cannot come to the hospital because my boss will not let me if I tell him that I'm going to the hospital every month*, *I'm going to lose my job”* (female HCW, clinical staff, FGD).

HCWs (6/9 FGDs) also mentioned that the implementation of Test and Treat is causing a rapid increase in the number of people coming to health facilities seeking ART services. Health facilities are at or over capacity to deal appropriately with HIV positive clients, causing additional delays for clients needing to navigate through several cares and counseling services on the same day.

### Facilitators to initiating ART

Several facilitators were mentioned by HCWs and clients, although facilitators were mentioned less frequently than barriers.

#### Staying healthy

HCWs (8/9 FGDs) noted that women were motivated to stay healthy in order to guarantee their children’s well-being. Most female clients (36/43) perceived ART as a major positive determinant of their own well-being and short- as well as long-term survival. Men also emphasized that starting ART early is a determinant of long-term survival (28/37), helping them to be healthy and prevent opportunistic infections. A few men mentioned the need to continue feeling healthy in order to work and make a living:

*“I like what I do*, *so I didn’t want to have to give up because I’m sick*, *so the concern was to start the treatment being healthy and to continue my work”* (Male client, Initiate, Maputo).

#### Health service models

Health providers (9/9 FGDs) believe that new strategies for HIV service delivery can improve ART uptake. Several new approaches to service delivery were suggested by HCWs, such as the adaptation of counseling sessions to clients’ specific needs (e.g. providing counselors with talking points that can be tailored to a client’s specific situation); locally-designed strategies to manage client flow and reduce waiting time in light of increased demand under Test and Treat, such as additional dedicated time for ART services and increased workforce; gender-specific consultations for mothers, children, and men; and ‘one-stop’ services with integrated HIV/primary care. Integrated services (HIV and primary care services) were perceived as particularly important because the strategy could decrease wait time and improve client privacy. In addition, HCWs believed community health-information programs were valuable resources to increase knowledge of ART, promoting timely treatment acceptance by clients.

Providers also noted that being professional, expressing empathy, having a caring attitude and investing time in counseling clients, were key strategies to facilitate the initiation of ART. Some clients (32/80) expressed feeling encouraged to continue accessing HIV services due to the supportive attitude of some HCWs:

*“Whenever I come here I don’t find the same doctor or the same nurse*, *but they always receive me well*. *Even when I go to get tablets*, *they ask me [about] sadness*, *they tell me not to feel bad*. *They always talk to us*. *They care about a sick person and everyone*. *They do the job well”* (Female client, Initiate, Matola).

## Discussion

This study is one of the first to examine barriers and facilitators for ART initiation in the context of universal Test and Treat among asymptomatic non-pregnant/breastfeeding adults. We find that already established barriers to care remain a problem for newly diagnosed clients under the Test and Treat policy; traditional barriers continue to limit ART initiation, alongside new barriers for healthy clients where good health makes it difficult for clients to accept a positive status or treatment initiation. Dominant barriers identified by both clients and providers in this study include: (1) feeling healthy; (2) non-acceptance of a positive HIV status; (3) poor knowledge about ART; (4) fear of ART side effects; and (5) fear of disclosure and lack of partner involvement due to stigma and discrimination. Less dominant, but still important, barriers identified include reluctance to start ART for life and needing time to accept positive test results before initiating treatment.[17, 19, 30–35] Provider perspectives closely mirrored accounts from client participants. Similar to our findings, several studies have reported that the idea of feeling ‘healthy’ was linked to refusing to initiate ART. [18, 34, 36, 37]

Negative attitudes among some HCWs, combined with the lack of privacy at clinics, distance to access services, and time needed to access HIV care were cited as key barriers to starting ART among clients in our study. Lack of privacy in HIV and ART services has been associated with individuals avoiding health facilities for fear of inadvertent disclosure to others.[17, 31, 33] Poor treatment of HIV-infected clients has been noted in other studies, particularly among pregnant women.[15] It is notable that in our study, there was a mixed experience with some clients reporting poor treatment at clinics and others specifically raising kindness and support from HCWs as a facilitator to ART initiation, including variation within a single clinic. It is likely that chronic shortages in health care workers strain the ability of existing HCWs to provide compassionate, holistic care.

Our findings also point to the rapid increase in HCWs’ workload as an important consideration as more countries start to implement Test and Treat. The implementation of multi-month scripting (3- or 6-month scripts) could address this concern by decreasing the number of clients seen each month.[38, 39] Expanding staffing may also be needed to accommodate the influx of new clients. Studies have shown the negative impact of an increase in HCWs’ workload without corresponding increase in manpower, particularly in a context with limited health system infrastructures.[19, 40, 41]

At the community level, some providers and clients suggested that fear of stigma and discrimination affected clients’ health-seeking behaviors. Several studies have described how anticipated stigma and fear of stigma are associated with non-disclosure and represent important barriers to treatment initiation.[17–20, 30–32, 35, 36, 42–45] In addition, religious beliefs[44] and alternative healing systems or remedies[34, 36] have been reported as barriers to ART initiation. At least one study identified the concern that ART may affect livelihood if side effects limit an individual’s ability to carry out daily work.[34] We found that fear of side effects was a common theme among clients, and individuals who feel healthy have concerns that starting ART will reduce their quality of life and their ability to carry out daily activities, including work. Current first-line ART in Mozambique includes the non-nucleoside reverse transcriptase inhibitor efavirenz (EFV), which is associated with central nervous system (CNS) side effects;[46] however, these are typically most pronounced early after initiation and often improve over time. Education around possible side effects and availability of other ART regimens may reassure patients and help them remain on treatment and/or seek care if they don’t feel well on first-line EFV. Newer regimens using integrase-inhibitors may eventually replace EFV with fewer CNS side effects.[47]

Our study found that a few key facilitators positively influenced clients’ willingness to start and remain in treatment. These included better knowledge about the benefits of ART, the desire for good health, and the desire to remain healthy to be able to care for dependents. [35] The desire to appear healthy and avoid the stigma associated with AIDS-related symptoms has been linked to individuals seeking to test and starting ART in other settings.[37, 48] Other studies have highlighted that being able to continue to care for family dependents is central to clients’ decisions to start treatment.[4, 35]

Test and Treat marks a paradigm change from previous strategies addressing the HIV epidemic. With the increasing focus on early treatment to optimize long-term health and treatment as prevention, understanding the unique barriers to ART that healthy individuals face is an important step to ensuring the success of Test and Treat in Mozambique and similar contexts. Our data suggest that the following strategies may be helpful optimize treatment uptake and retention in Test and Treat among healthy clients:

- Individual and group counseling should include messages targeted to clients’ specific needs. Specific counseling material is needed about the benefits of starting on ART while feeling healthy and remaining on ART for life, as well as specific strategies healthy clients can use to overcome barriers to care. Such information may help clients better understand and accept immediate treatment initiation as part of Test and Treat.
- Community sensitization campaigns are needed to improve client understanding and acceptance of immediate treatment initiation under Test and Treat, even before they reach the health facility. Information on the benefits of treatment for prevention should also be included. Recent data show understanding treatment as a form of prevention may increase use of testing and treatment at the community-level.[49]
- Strategies to improve client privacy and decrease wait times should be pursued. Locally-designed strategies may help manage client flow and reduce wait times in light of increased demand under Test and Treat. ‘One-stop’ services with integrated HIV/primary care may also decrease concerns around client privacy and wait times.[50] Client-centered HIV services should continue to be pursued as they increase support and improve the over-arching environment in which care is delivered, potentially improving ART uptake and retention among healthy clients. Privacy may also be improved through community-based distribution strategies.
- Multi-month dispensing strategies are needed to accommodate increased demands for ART. Three-month scripting is well established and successful in other settings.[51, 52] Likewise, six-month scripting has been validated in other settings[38, 39] and is currently being evaluated in Malawi and Zambia, similar low-resourced settings as Mozambique.[53]
- Additional investments in staffing may be needed to support the increasing number of clients initiating ART. In particular, there may be a need for additional support staff (counselors and peer-educators) who can provide additional support to healthy clients initiating ART under Test and Treat.

The implementation of Test and Treat in Mozambique marks an important paradigm shift in the enrolment of clients in HIV care, with asymptomatic non-pregnant/breastfeeding adults now being offered ART. This study provides novel insights into the way previously established barriers and facilitators are exacerbated for healthy clients in the context of universal access to treatment. In order to optimize uptake and retention and achieve the 90/90/90 goals as established by UNAIDS, programs will need to address barriers for this population, including stigma reduction, improved counseling and appropriate education on HIV services, emphasis on compassionate, client-centered care, and improved privacy and confidentiality at ART clinics. The implementation of the Test and Treat guidelines has generated a rapid increase in the number of clients enrolled in HIV care, and in the perceived workload of HCWs. New strategies to improve efficiencies and potentially expand the workforce may help to reduce barriers to care, improve uptake and retention, and optimize long-term outcomes for clients.

### Limitations

This study included the perspectives of health providers, as well as men and women who have both accepted and refused ART as part of the national Test and Treat program; however, there are several limitations. First, over half of respondents were diagnosed between 3–6 months prior to completing the in-depth interview. This may create recall bias. However, findings were similar with clients who were diagnosed recently (<3months), suggesting recall bias is minimal. Second, the cross-sectional nature of the research design means that we are unable to confirm that perceived barriers and facilitators predict ART initiation (or not). Finally, representation of findings may be limited as only 4 of Mozambique’s 10 provinces were included in the study, and we excluded provincial and private hospitals, as well as smaller, rural health facilities, where perspectives of HIV-infected clients and HCWs may be different.
